# Availability and stock-out duration of essential medicines in Shegaw Motta general hospital and Motta Health Centre, North West Ethiopia

**DOI:** 10.1371/journal.pone.0274776

**Published:** 2022-09-16

**Authors:** Bereket Bahiru Tefera, Chernet Tafere, Adane Yehualaw, Ephrem Mebratu, Yalelet Chanie, Simachew Ayele, Sewnet Adane

**Affiliations:** 1 Department of Pharmacy, College of Medicine and Health Science, Bahir Dar University, Bahir Dar, Ethiopia; 2 Department of Pharmacy, College of Health Science, Debre Markos University, Debre Markos, Ethiopia; Flinders University, AUSTRALIA

## Abstract

Essential medicines are those that meet the population’s most pressing healthcare needs. As a result, they should always be available in sufficient quantities and the proper dosage forms within health facilities. The study aimed to determine the availability and stock-out duration of essential medicines in Shegaw Motta General Hospital and Motta Health Centre. Descriptive study was conducted at Shegaw Motta General Hospital and Motta Health Centre. The data were collected from June-20 to July-20, 2021 G.C. Fifteen essential medicines from both health facilities’ tracer drug lists were reviewed. The frequency and percentage of availability and stick-out duration were calculated, and the results were briefly described in the text and displayed in tables. The average availability of essential medicines on the day of the survey was 80% and 93.3% in Shegaw Motta general hospital and Motta health center, respectively. Besides, 60% and 20% of the essential medicines were stocked out at least once in the last six months (before the data collection period) in the hospital and health center, respectively. The average stock-out duration in the last six months period (before the data collection period) was 38.8 days (ranges from 10 days to 157 days) in the hospital and 11.2 days in the health center. Ferrous salt + folic acid 150mg + 0.5mg and Medroxy Progesterone injection were the medicines with the longest stock-out duration in Shegaw Motta general hospital and Motta health center, respectively.The average availability of essential medicines was fairly high both in the hospital and health center. In comparison to the hospital, the health center had better availability and a shorter stock-out duration. Over the last six months, a significant percentage of essential medicines were stocked out at the hospital.

## Introduction

Essential medicines (EM) are those that meet the population’s healthcare needs and are intended to be available at all times in the context of a functioning healthcare system in adequate quantities, in the appropriate dosage forms, and at a price that the community can afford [1]. Preparing a comprehensive national essential medicines list is recommended by WHO for all countries to improve access to safe, effective, and high-quality medicines [2]. National essential medicine lists are government approved lists of medicines, often adapted and implemented as local medicine formularies [3]. Based on WHO directions, Ethiopia developed its own national essential medicine list for use in healthcare financing and medicines supply budgeting; procurement, and encouraging the appropriate use of medicines [4].

The WHO estimated that health interventions based on the delivery of EMs could prevent over 10 million deaths each year [2]. Developing an essential medicines policy (EMP) can help countries rationalize the purchase and distribution of medicines, thereby reducing the expenditures in public and private health systems, improving their accessibility, and enhancing their rational use [5]. Consumers frequently cite the availability of essential medicines as the most important aspect of quality healthcare services, and a shortage of medicines is a major factor in the underutilization of government health services [6]. The continual availability of essential medicines can provide patients with value-added services [7].

The provision of quality health services can be adversely affected by a stock-out of even a small number of essential medicines. Health service readiness is impacted by stock-outs, since clinics without consistent and adequate availability of essential medicines cannot provide quality care [8]. It is essential to understand the degree, reasons for, and implication of stock-outs of essential medicines at different healthcare levels in order to optimize quality of care. Approximately one-third of the world’s population lacks regular access to full and effective treatment with the necessary medicine [9, 10]. More than 33% of the world’s population and 50% of people in the poorest countries in Africa and Asia lack access to essential medicines [11]. An effective healthcare system should ensure equitable access to essential medicines, vaccines, and healthcare technologies that are of guaranteed quality, safety, efficacy, and cost-effectiveness profile [12].

Healthcare policy and system flaws lead to inequities in access to medicines [1]. There are several factors that can contribute to the stockout of EMs, including limited budget allocation, inefficient budget utilization, irrational demand estimation, poor distribution systems, supplying medicines with short shelf lives, and purchasing unnecessary medicines [13]. The ability and practice of the store clerk also affect the stock status [12, 14, 15]. Public procurement inefficiency, limited budgets, and fluctuating currency exchange rates are some of the factors that contribute to a shortage of medicines in Ethiopia. Besides, Medicine availability in Ethiopia is hindered by a lack of transportation infrastructure and long distances from suppliers, especially in rural areas where medicines are sometimes transported by humans [14–17]. The lack of resources or information can create barriers to accessing essential medicines in many developing countries, contributing to higher rates of death and illness [18].

The Pharmaceutical Fund and Supply Agency (PFSA) was established in Ethiopia in order to ensure that medicines were available, fix price variations, and eliminate interruptions in supply. To supply pharmaceuticals to Ethiopia’s public health facilities, PFSA designed and implemented an integrated pharmaceutical logistics system [17, 19]. to Even with significant progress since the introduction of the EMs concept, access to essential medicines remains a serious public health issue [20]. When medicine shortage happen in the public sector, patients are forced to purchase medicines from private outlets, where generic medicines cost many times more than the international reference price, and originators are often even more expensive [13]. Lack of EMs and high spending on low-quality medicines result in resource wastage and, in the worst-case scenario, death [21]. Our study’s goal was to assess the availability and stock-out duration of essential medicines in selected public healthcare facilities.

## Materials and methods

### Study area and period

The research was conducted at Shegaw Motta General Hospital and Motta Health Center, both located in the town of Motta. Motta town is located 371 kilometers from Bahir Dar (the capital of Amhara Regional State) and 450 kilometers from Addis Ababa. The hospital served an estimated 1.2 million residents around the area, while the health center was also providing services for an estimated 250 thousand people. In Motta town, there is only one general hospital and one health center. These are the only health facilities in the area providing basic health care to the residents. The hospital serve as referral site for cases that cannot be managed by health center. The Data was collected from June 20 to July 20, 2021 G.C.

### Study design

We conducted an institution-based descriptive study to assess the availability and stock-out duration of essential medicines.

### Source population

Source populations included all essential medicines at Motta health center and Shegaw Motta general hospital, all bin card documents of medicines, pharmaceutical store managers at both health institutions.

### Study population

This study included selected essential medicines, the bin cards of selected medicines, a pharmaceutical store manager of each health facilities as the study populations.

### Inclusion criteria and exclusion criteria

In this study, only essential medicines appearing on the tracer drug list of the hospital and health center were selected. To determine availability and stock-out duration, we reviewed bin-card transactions of the last 6 months (prior to the data collection period). The study excluded medical supplies, equipment, reagents, and essential medicines without a bin-card.

### Sample size determination

A total of fifteen essential medicines that were identified in the tracer drug lists of both health facilities, were assessed to determine the availability and stockout duration of essential medicines. One pharmaceutical store manager at each of the health facilities was also interviewed to collect demographic information.

### Sampling technique

The pharmaceutical store managers were chosen purposely since they were the most accessible staff for data collectors. As well, every essential medicine found in the tracer drug lists of both health facilities (a total of fifteen drugs were found in both lists) was selected for evaluation. Tracer drug lists should be prepared by health facilities based on tracer drugs lists prepared by the Ethiopian Federal Minister of Health. Tracer drugs were selected based on their usefulness in treating or diagnosing the most common diseases [22]. To review the availability of specific medicines, one bin-card was selected for each selected medicine (which means 15 bin-cards total).

Essential medicines evaluated in this study include Amoxicillin 500 mg, Artemether + lumefantrine 20mg + 120mg, Ceftriaxone 1gm injection, Cotrimoxazole 240mg/5ml suspension, Ferrous salt + folic acid 150mg + 0.5mg tablet, Gentamycin 40mg/5ml injection, Hydralazine injection, Magnesium Sulphate injection, Oral Rehydrate Salt (ORS), Oxytocin injection, Pentavalent vaccine, Rifampicin/Isoniazid/Ethambutol/Pyrazinamide fixed-dose combination, Med Roxy progesterone injection, Tetracycline 1%, and Tenofovir + Lamuvidine + Efaverenz Adult dose.

### Data collection tool and procedure

To collect data, a structured questionnaire was prepared in English. This questionnaire was adapted from the Logistics Indicators Assessment Tool developed by USAID DELIVER [23]. We reviewed the bin-card records of each selected medicine for the past six months to measure its availability and stock-out duration. These data were collected by pharmacy personnel with expertise in medicines management.

### Data processing and analysis

The data were evaluated for completeness and analyzed using Microsoft Office Excel. We computed descriptive statistics (frequency and percentage) and summarized the results with texts and graphs. We analyzed and presented the data separately for each health facility.

### Operational definition

Availability refers to the physical availability of non-expired and usable medicines in a hospital’s inventory. We have classified availability as follows: less than 30% as "Very Low", 30% - 49% as "Low", 50% - 80% as "Fairly High", and greater than 80% as "High" [17].

### Ethical consideration

The National Research Ethical Review Guideline for Ethiopia allows a study to be exempt from ethical review if its purpose is to study government programs designed to provide public services and the investigator collected the information in a way that cannot identify the study participants. All respondents verbally consented before data collection commenced, and their confidentiality was assured. As the study carries only minor risks for participants, verbal informed consent is considered acceptable by the National Research Ethical Review Guideline [24].

## Result

### General characteristics of health facilities

The health facilities included in the study were a general hospital and a health center. Each facility had an average lead time of one month. There were 9 and 6 medicine dispensing units in the hospital and health center, respectively. There were 12 pharmacy staff members at the hospital and 4 at the health center. The pharmacist was in charge of the hospital’s medicine store. The health center’s medicine store, on the other hand, was run by a druggist. (Table 1).

**Table 1:** General characteristics of health facilities.

NO	Characteristics	SMGH*	MHC**
1	Type of health facilities	General hospital	Health centre
2	The average Lead time of the health facility	1 month	1 month
3	Number of medicines dispensing units	9	6
4	Number of pharmacy staff	12	4
5	Numbers of medicines store manager	1	1
6	Academic status of medicine store managers	Pharmacist	Druggist

*Shegaw Motta General Hospital

** Motta Health Centre

### Availability and stock out of essential medicines in Shegaw Motta general hospital and Motta Health Centre

On the day of the survey, only 80% (12) of the 15 essential medicines were available at the Shegaw Motta general hospital and 93.3% (14) were available at the Motta health center. During the last six months, 60% (9) of essential medicines were stocked out at Shegaw Motta general hospital while only three (20%) were stocked out at Motta health center. (Table 2)

**Table 2:** Availability of essential medicines on the day of the survey and in the last 6-month before data collection.

**Health institution**	**On the day of the survey**		
		Frequency (N = 15)	Percent (%)
Shegaw Motta General Hospital	Available	12	80
	Not-available	3	20
Motta health center	Available	14	93.3
	Not-available	1	6.7
**Health institution**	**Stockout at least once in the last 6 months before data collection**		
		Frequency (N = 15)	Percent (%)
Shegaw Motta General Hospital	Available	6	40
	Not-available	9	60
Motta health center	Available	12	80
	Not-available	3	20

During our survey day, in Shegaw Motta general hospital, three essential medicines were unavailable, including Amoxicillin 500mg, Cotrimoxazole 240mg/5ml, and Ferrous salt + folic acid 150mg + 0.5mg. Furthermore, every essential medicine except Medroxy progesterone injection was available in the Motta health center. (Table 3)

**Table 3:** Availability of each medicine in Shegaw Motta general hospital and Motta Health center at the date of the survey.

Essential medicine	Motta Health Centre		Shegaw Motta General Hospital	
	Available	Not-available	Available	Not-available
Amoxicillin 500 mg	*			*
Artemether + lumefantrine 20mg + 120mg	*		*	
Ceftriaxone 1gm injection	*		*	
Cotrimoxazole 240 mg/5ml	*			*
Ferrous salt + folic acid 150 mg + 0.5mg	*			*
Gentamycin injection 40mg/5ml	*		*	
Hydralazine injection	*		*	
Magnesium Sulphate injection	*		*	
Medroxy progesterone injection		*	*	
Oral Rehydrated Salt (ORS),	*		*	
Oxytocin injection	*		*	
Pentavalent vaccine	*		*	
Rifampicin/isoniazid/ethambutol/pyrazinamide fixed dose combination	*		*	
Tetracycline 1%	*		*	
TDF+3TC+EFV adult dose	*		*	

### Stock-out duration of essential medicines in Shegaw Motta general hospital and Motta Health Centre

The average number of stock-out days at Shegaw Motta general hospital was 38.5 days. Over the last six months, Ferrous salt + folic acid 150 mg + 0.5 mg was the medicine with the most stocked out days (157 days), followed by Tetracycline 1%, and Oral Rehydration Salt with 127 days, and 104 days, respectively. In Motta health center, only three medicines were not available during the last six months and the average stock-out days was 11.2 days. In terms of stockout days among these medicines, Medroxy progesterone injection ranked highest with 150 days. And it was followed by Ferrous salt + folic acid 150mg + 0.5mg which was stocked out for 12 days in the last 6-months. (Table 4)

**Table 4:** Stock-out duration of essential medicines in the last 6-months in Shegaw Motta general hospital and Motta health center.

Essential medicines	Stock-Out Duration In Days	
	Shegaw Motta General Hospital	Motta Health cetre
Amoxicillin 500 mg	40	0
Artemether + lumefantrine 20mg + 120mg	0	0
Ceftriaxone 1gm injection	57	0
Cotrimoxazole 240mg/5ml	50	0
Ferrous salt + folic acid 150mg + 0.5mg	157	12
Gentamycin injection 40mg/5ml	0	0
Hydralazine injection	0	0
Magnesium sulfate injection	0	0
Medroxy progesterone injection	0	150
Oral Rehydrated Salt (ORS),	104	0
Oxytocin injection	16	0
Pentavalent vaccine	0	0
Rifampicin/isoniazid/ethambutol/pyrazinamide fixed dose combination	17	0
Tetracycline 1%	127	6
TDF+3TC+EFV adult dose	10	0

## Discussion

On the day of the survey, The availability of selected EMs in both hospital and health center was 80% and 93.3% respectively. At both health facilities, availability was below the WHO recommendation of 100%, but at the health center, it was above the minimum requirement of WHO (80%). The availability at Shegaw Motta general hospital was comparable to that of a study in Tanzania, while the availability at Motta health center was less than that of the Tanzanian study [25]. The availability found in this study was much higher than what was reported in a study conducted in developing and middle-income countries [26]. It may be because the findings of the latter study were based on the findings of many health facilities, whereas this study reported findings from two separate health facilities. In addition, the report in this study was higher than the report in Gondar [27]. A study conducted in Jimma reported 55.65% availablity of essential medicines, which was much lower than availability at both Shegaw Motta general hospital and Motta health center [17]. As the studies were done on different period, this disparity may reflect an improvement in access to essential medicines in Ethiopia compared to past years.

At the hospital and health center, respectively, 60% and 20% of essential medicines were stocked out during the last six months. As compared to a study conducted in Gondar, the findings of the Shegaw Motta general hospital were significantly higher, whereas those of Motta health center were equivalent [27]. The provision of essential medications is crucial for meeting the most basic needs of the people, as well as promoting the rational use of medicines to improve the effectiveness of health resources [28]. As part of activities to increase the availability of EMs, the government should promote competition for lower-cost generics, purchase generics at lower prices that are of high quality, negotiate prices with suppliers, eliminate stockouts by forecasting adequately, and secure adequate funding [29].

Over the last six months, the average stock-out duration at Shegaw Motta general hospital and Motta health center was 38.5 days and 11.2 days, respectively. Furthermore, the average stockout period in Shegaw Motta general hospital during the last six months was slightly longer than what was found in the Gondar study. However, the average stockout period in Motta health center was shorter than that in the Gondar study [27]. Among the medicines in the Shegaw Motta general hospital, ferrous salt + folic acid 150 mg + 0.5mg had the longest stockout period, with 157 days. In Motta health center, the medicine with the longest stockout days was Medroxy progesterone injectable, which was unavailable for 150 days. The shortage of essential medicines can increase the workload of already stressed healthcare workers, since patients are faced with additional visits and referrals. This frequent stockout is likely due to long-distance transportation from its suppliers during the procurement process, as well as the unavailability of pharmaceuticals from suppliers [8, 30]. It is possible that the COVID-19 pandemic may compromise access to essential medicines and medical supplies, which may impact prevention, diagnosis, care, and treatment [22]. In addition, the government’s limited funds for medicine procurement and inadequate pharmaceutical management in these facilities may contribute to frequent medicines stockouts [31].

### Study limitation

The study only evaluated selected essential medicines, but assessing all medicines in health facilities would be more meaningful. This study only examined the availability status at two health facilities; an evaluation of the situation at other facilities in the vicinity would be more representative. Moreover, the study did not explore any of the factors that affect the availability of essential medicines in health care facilities.

## Conclusion

In general, the hospital tends to have lower drug availability and a higher history of stockouts than the health center. Besides, at least one of the essential medicines was not available in both health facilities on the day of the survey. Similarly, in the six months before the survey, majority of essential medicines were out of stock in the hospital. The average stock-out duration at Shegaw Motta general hospital was longer than that og the helth center.

### Recommendations

It is necessary for both health facilities to work on improving the availability of some essential medicines since some items have been unavailable for more than four months. Achieving the maximum possible availability of essential medicines is a goal that should be pursued by different stakeholders in the Ethiopian healthcare system. In order to mitigate the stockout and launch strategic solutions, it will be necessary to identify and analyze possible factors that affect availability of essential medicines. It is imperative that Ethiopia’s government, academic institutions, and other researchers take this gap into account in the future, since this study didn’t address it. As well, it would be worthwhile to assess the availability of medicines at nearby area health facilities because patients may also travel there for care.

## Supporting information

S1 Table Stock-out duration in days within the last 6 months. (XLSX)

S2 Table Availability data of essential medicines. (XLSX)
